# The use of endoscopic ultrasound in tandem with endoscopic retrograde cholangiopancreatography in the 2019 American Society for Gastrointestinal Endoscopy guideline for patients at high risk of choledocholithiasis can help to avoid diagnostic endoscopic retrograde cholangiopancreatography in individuals without ascending cholangitis

**DOI:** 10.1002/deo2.70058

**Published:** 2025-01-22

**Authors:** Apichet Sirinawasatien, Jiravit Chanchairungcharoen, Thanapon Yaowmaneerat, Jirat Jiratham‐opas, Kanokpoj Chanpiwat, Tanyaporn Chantarojanasiri, Siriboon Attasaranya, Kannikar Laohavichitra, Jerasak Wannaprasert, Thawee Ratanachu‐ek

**Affiliations:** ^1^ Department of Medicine Rajavithi Hospital College of Medicine Rangsit University Bangkok Thailand; ^2^ Department of Medicine Nanthana‐Kriangkrai Chotiwattanaphan Institute of Gastroenterology and Hepatology Faculty of Medicine Prince of Songkla University Songkhla Thailand; ^3^ Department of Surgery Hatyai Surgical Endoscopic Center Hatyai Hospital Songkhla Thailand; ^4^ Department of Surgery Rajavithi Hospital College of Medicine Rangsit University Bangkok Thailand

**Keywords:** ascending cholangitis, choledocholithiasis, common bile duct stone, endoscopic retrograde cholangiopancreatography, endoscopic ultrasound

## Abstract

**Objectives:**

Choledocholithiasis is the leading cause of biliary pancreatitis and biliary sepsis. Endoscopic retrograde cholangiopancreatography (ERCP) is considered a minimally invasive treatment for choledocholithiasis. However, diagnostic ERCP should be avoided. We conducted a prospective trial in high‐risk choledocholithiasis patients based on the American Society for Gastrointestinal Endoscopy (ASGE) 2019 criteria to investigate the diagnostic accuracy of the current guideline.

**Methods:**

This multicenter, prospective cohort study included 240 consecutive patients. The primary outcome was the performance of the criteria in predicting choledocholithiasis. The secondary outcome was a percentage reduction in diagnostic ERCP when endoscopic ultrasound was used in tandem with ERCP in individuals without ascending cholangitis.

**Results:**

The overall criteria revealed a positive common bile duct (CBD) stone in 87.1% of patients. Regarding the diagnostic performance of each criterion, ascending cholangitis had a specificity of 67.7% and a positive predictive value (PPV) of 90.2%; total bilirubin >4 mg/dL and dilated CBD had a specificity of 74.2% and a PPV of 55.6%; and CBD stone on ultrasound/cross‐sectional imaging had a specificity of 58.1% and a PPV of 89.2%. Of the 138 patients without ascending cholangitis who met the other two high‐risk criteria and were sent for EUS first, 21 cases (15.2%) were able to avoid a diagnostic ERCP.

**Conclusions:**

The current ASGE 2019 criteria yield acceptable choledocholithiasis diagnostic accuracy. Using endoscopic ultrasound to confirm CBD stones before ERCP can help almost half of patients with the specific condition of total bilirubin >4 mg/dL and dilated CBD to avoid diagnostic ERCP.

## INTRODUCTION

Gallstones are a common gastrointestinal condition that affects 10%–15% of the general population,^1^ with up to 20% of patients with symptomatic gallstones possessing coexisting bile duct stones (choledocholithiasis).^2^ Moreover, when it remains untreated, choledocholithiasis is the leading cause of biliary pancreatitis and biliary sepsis.^3^, ^4^ Endoscopic retrograde cholangiopancreatography (ERCP) is considered a minimally invasive treatment for choledocholithiasis.^5^ However, since substantial, serious complications can occur post‐procedurally, the most important early adverse event associated with ERCP is post‐ERCP pancreatitis, which is potentially fatal and could develop up to 10%; diagnostic ERCP should be avoided when possible.^6^

The American Society of Gastrointestinal Endoscopy (ASGE) published guidelines in 2010,^7^ updated in 2019,^8^ for stratifying patients into high (>50%), intermediate (10%–50%), or low‐risk (<10%) categories for choledocholithiasis. High‐risk patients should proceed directly to ERCP, while intermediate‐risk patients should undergo a confirmatory test, such as endoscopic ultrasound (EUS), magnetic resonance cholangiopancreatography (MRCP), or intraoperative cholangiogram (IOC), prior to ERCP. Ascending cholangitis is one of the three high‐risk ASGE 2019 criteria, warranting ERCP for diagnostic and therapeutic purposes. The other criteria—a common bile duct (CBD) stone detected on ultrasound (US) or cross‐sectional imaging, or total bilirubin (TB) >4 mg/dL with a dilated CBD on the US or cross‐sectional imaging—may yield false negatives due to spontaneous stone passage or because of another obstruction. To minimize unnecessary ERCP, EUS can confirm CBD stones before proceeding with stone clearance in the same endoscopic session. Despite the fact that diagnostic EUS for choledocholithiasis has a low but finite risk of perforation (0.02%–0.07%),^9^ the procedure should only be performed by an experienced endoscopist.

In a prospective trial, we evaluated patients with suspected choledocholithiasis based on ASGE 2019 high‐risk criteria, implementing an EUS‐ERCP model. Patients without ascending cholangitis (i.e., CBD stone or TB >4 mg/dL with a dilated CBD on imaging) underwent EUS first, followed by therapeutic ERCP if choledocholithiasis was confirmed. Those with ascending cholangitis proceeded directly to ERCP. The trial assessed the ASGE guidelines' accuracy in predicting choledocholithiasis and the model's role in reducing diagnostic ERCP.

### Patients and methods

#### Study design

This multicenter prospective cohort study, conducted from August 2021 to November 2023 at three tertiary hospitals in Bangkok and Songkhla, Thailand, evaluated the diagnostic performance of ASGE 2019 high‐risk criteria for predicting choledocholithiasis and the role of EUS in reducing unnecessary ERCP. The primary outcome was the criteria's accuracy in confirming choledocholithiasis via EUS or ERCP, while the secondary outcome measured the reduction in diagnostic ERCP when EUS was used in patients without ascending cholangitis. The study followed the Declaration of Helsinki's ethical guidelines, and the protocol was reviewed and approved by the ethics committee of Rajavithi Hospital (approval no. 026/2564). Written informed consent was obtained from the patients prior to study enrollment. The study was registered with the Thai Clinical Trials Registry (registration no. TCTR20210812001).

#### Sample size

Based on institutional ERCP data from 2018 to 2020, with an annual choledocholithiasis prevalence of 33% among ∼1000 procedures, the sample size was calculated using a one‐proportion estimation formula for 80% power and a 2‐sided *p*‐value <0.05. A required sample of 197 was increased to 240 to account for a 20% dropout rate.

#### Patients

The inclusion criteria were individuals aged 18–80 suspected of having choledocholithiasis based on the high‐risk ASGE 2019 criteria below^1^: ascending cholangitis as defined by the 2018 Tokyo guidelines criteria,^2^ ^,10^ a CBD stone on US or cross‐sectional imaging, or^3^ a TB >4 mg/dL with a dilated CBD on US/cross‐sectional imaging.

The exclusion criteria were as follows^1^: history of liver transplantation,^2^ decompensated cirrhosis defined by Child‐Turcotte‐Pugh score >9,^3^ preexisting hepatobiliary diseases, such as biliary stricture, active viral hepatitis, or alcoholic hepatitis,^4^ history of biliary surgery,^5^ suspicions of pancreaticobiliary cancer, and^6^ a previously placed endo‐biliary stent or sphincterotomy.

#### Abdominal ultrasonography or cross‐sectional imaging and data collection

Patients were eligible if they had symptomatic ascending cholangitis, suspected choledocholithiasis (epigastric or right upper quadrant abdominal pain with abnormal liver tests), or asymptomatic findings from a health check‐up and underwent abdominal US, computed tomography (CT), or MRCP. CBD dilation was defined as a duct diameter > 6 mm for those with gallbladders and > 8 mm post‐cholecystectomy. Baseline characteristics, imaging, and initial laboratory results were collected to assess risk.

#### Endoscopic procedures

**FIGURE 1:**
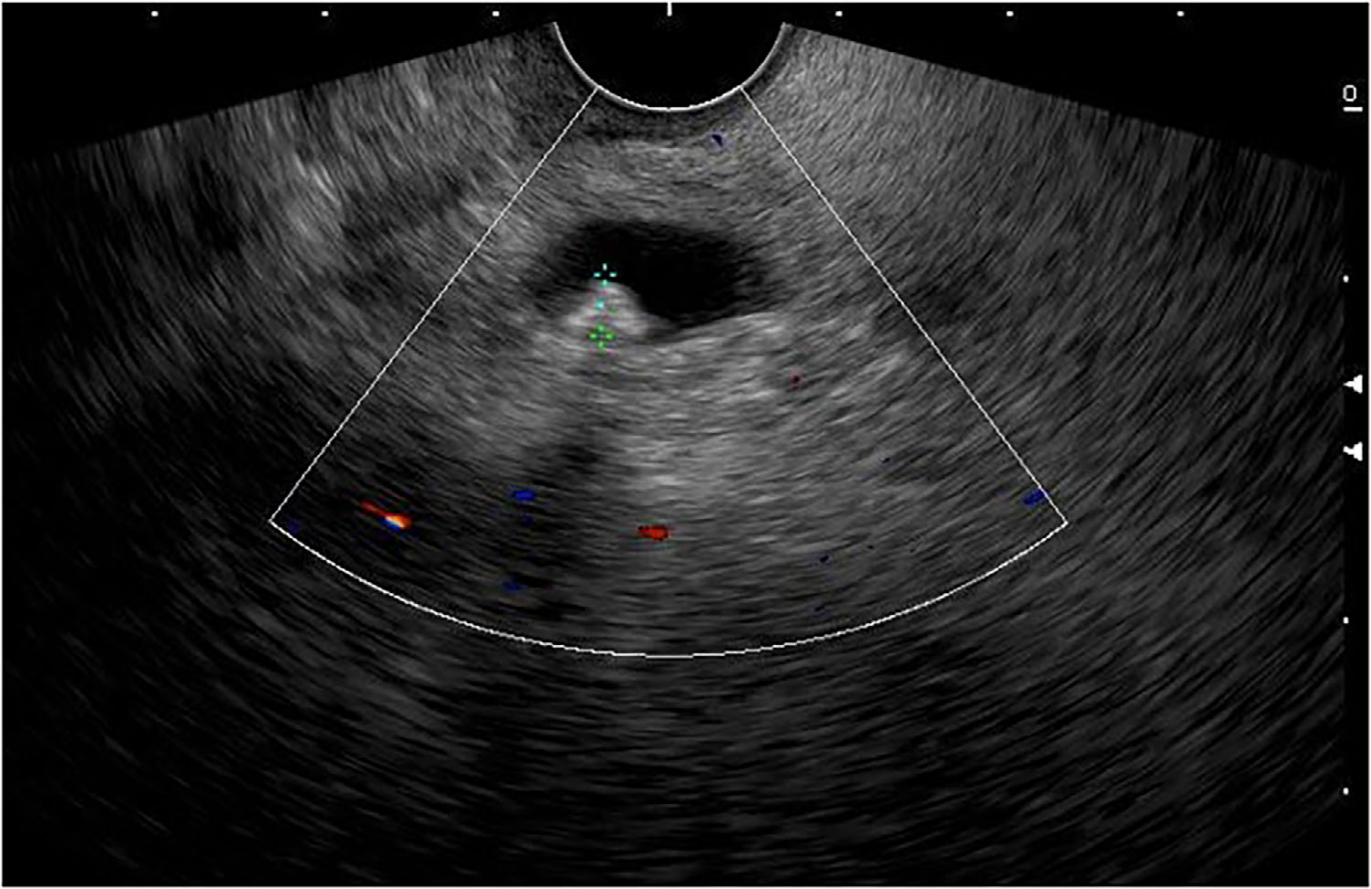
An endoscopic ultrasound (EUS) image revealing a single stone in a dilated common bile duct.

Patients with ascending cholangitis underwent ERCP to determine the underlying cause of CBD obstruction. Sphincterotomies and stone extractions were performed if CBD stones were identified using standard techniques using a duodenoscope (Model TJFQ‐190 V; Olympus Optical Corporation or Model ED‐580XT; Fujinon Corporation). Patients with a CBD stone on US/cross‐sectional imaging or a TB >4 mg/dL with a dilated CBD on US/cross‐sectional imaging initially underwent EUS. All EUS procedures were performed by one of six experienced endosonographers (Siriboon Attasaranya, Jirat Jiratham‐opas, Tanyaporn Chantarojanasiri, Apichet Sirinawasatien, Kannikar Laohavichitra, and Thawee Ratanachu‐ek; each with experience performing more than 1000 EUS examinations for pancreatobiliary diseases). The EUS examinations were performed with an electronic radial or linear EUS scope by Olympus (Model GF‐UCT 180; Olympus Optical Corporation) with Aloka Arietta 850 ultrasound processor or Fujinon (Model EG‐580 UT; Fujinon Corporation) with SU‐1‐H ultrasound processor. CBD stones were defined as echogenic material or hyperechoic lesions, with or without posterior acoustic shadow on EUS (Figure 1). If CBD stones were present, the patients underwent ERCP for stone extraction in the same session of EUS.

Since follow‐up imaging is impractical for our setting, we assess CBD stone clearance by monitoring clinical symptoms and liver biochemical tests during outpatient visits over a minimum of 6 months. Patients without recurrent abdominal pain and with normal liver biochemical values are considered to have a low (<10%) likelihood of choledocholithiasis,^7^ and in such cases, the risk and cost of EUS do not justify further investigation, making expectant management a more favorable approach. However, if clinical symptoms or liver test abnormalities indicate possible symptomatic cholelithiasis, EUS is performed to confirm the presence of CBD stones.

#### Statistical analysis

Statistical analyses were performed using SPSS version 17.0 (SPSS Inc.). Descriptive statistics summarized demographic and baseline data, with continuous variables reported as mean ± SD or median (interquartile range [IQR]) and categorical variables as numbers and percentages. A 2 × 2 table calculated the diagnostic accuracy, sensitivity, specificity, positive predictive value (PPV), negative predictive value (NPV), and 95% confidence intervals (CIs) of the ASGE 2019 high‐risk criteria for choledocholithiasis. Statistical significance was set at *p*‐values < 0.05.

## RESULTS

**FIGURE 2:**
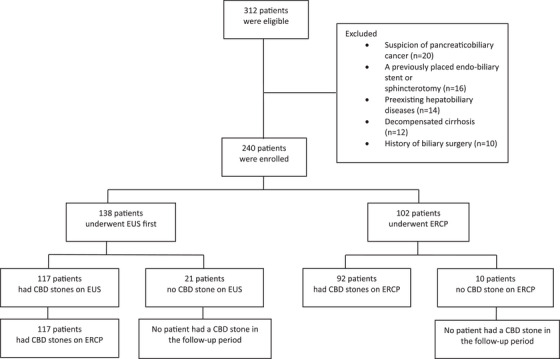
Study Flowchart. A total of 312 patients met the inclusion criteria, of whom 72 were excluded. Thus, 240 patients were enrolled: 102 with ascending cholangitis who underwent ERCP, and 92 with CBD stones, while 10 had no CBD stones. Additionally, 138 patients without ascending cholangitis underwent EUS. Of these, 117 were diagnosed with CBD stones by EUS and underwent ERCP, while 21 had no CBD stones on EUS and were followed clinically with no recurrence of CBD stones. EUS, endoscopic ultrasound; ERCP, endoscopic retrograde cholangiopancreatography; CBD, common bile duct.

The study flowchart (Figure 2) shows 138 patients undergoing initial EUS, with 117 proceeding to ERCP after CBD stones were detected; stones were confirmed in all 117 cases. Among 21 EUS‐negative patients, none developed CBD stones during follow‐up. Of 102 ascending cholangitis patients undergoing direct ERCP, 92 (90.2%) had CBD stones, while 10 (9.8%) did not, with no subsequent stone detection in follow‐up.

### Baseline characteristics

The study included 240 patients, with ascending cholangitis in 42.5%, TB >4 mg/dL with a dilated CBD in 7.5%, and CBD stones on US/cross‐sectional imaging in 50% based on ASGE 2019 high‐risk criteria. The mean age was 63.2 years (standard deviation [SD] 16.5), and 44.8% were male. Prior cholecystectomy was reported in 11.3%, upper abdominal pain was the most common symptom (87.9%), and acute gallstone pancreatitis occurred in 3.8%. Among 138 patients without ascending cholangitis, 80.4% (111 patients) presented with upper abdominal pain, 31.2% (43 patients) had jaundice, and 6.5% (9 patients) were asymptomatic. CBD stones were detected in 187 patients (77.9%) on US or cross‐sectional imaging (mostly CT scans), with a median size of 9 mm (IQR 6–12 mm).

Among 120 patients classified as high‐risk per ASGE 2019 criteria due to CBD stones on imaging, 27.5% were detected via abdominal ultrasound, with a median stone size of 13 mm (IQR 10–16 mm). Cross‐sectional imaging, comprising CT (66.7%) and MRCP (5.8%), identified stones with a median size of 8 mm (IQR 5–11 mm). The median time from initial presentation to endoscopic procedure, either EUS or ERCP, was 6 days (IQR 2–15 days; Table 1).

**TABLE 1:** Baseline characteristics of the study population (*N* = 240).

Age, mean (SD)	63.2 (16.5)
Male sex, *n* (%)	110 (45.8)
Body mass index (kg/m^2^), mean (SD)	24.5 (4.9)
Underlying diseases, *n* (%)	
▪ HT	112 (46.7)
▪ DM	73 (30.4)
▪ DLP	86 (35.8)
▪ CVD	38 (15.8)
▪ None	56 (23.3)
Prior cholecystectomy, *n* (%)	27 (11.3)
Presenting symptoms of all patients, *n* (%)	
▪ Upper abdominal pain	211 (87.9)
▪ Temperature ≥38°C	101 (42.1)
▪ Ascending cholangitis	102 (42.5)
▪ Charcot's triad	33 (13.8)
Presenting symptoms of the patients without ascending cholangitis, *n* (%)	138 (57.5)
▪ Upper abdominal pain	111 (80.4)
▪ Jaundice	43 (31.2)
▪ Asymptomatic	9 (6.5)
Acute Pancreatitis, *n* (%)	9 (3.8)
Laboratory test values, median (IQR)	
▪ Hemoglobin (g/dL)	12.3 (11.2–13.2)
▪ WBC count x 10^9^/L	9850 (6865–13,925)
▪ AST (U/L)	143 (56–282)
▪ ALT (U/L)	145 (53–309)
▪ Alkaline phosphatase (U/L)	196 (122–318)
▪ Total bilirubin (mg/dL)	3.2 (1.4–5.5)
▪ Directed bilirubin (mg/dL)	2.2 (0.8–3.8)
▪ Albumin (mg/dL)	3.8 (3.5–4.1)
▪ Globulin (mg/dL)	3.6 (3.2–4.0)
Ultrasound/cross‐sectional imaging, *n* (%)	
▪ Dilated CBD	117 (48.8)
▪ CBD stone	187 (77.9)
▪ CBD stone size, millimeter, median (IQR)	9^6^, ^7^, ^8^, ^9^, ^10^, ^11^, ^12^
Patients with the ASGE 2019 high‐risk criteria, *n* (%)	
▪ Ascending cholangitis	102 (42.5)
▪ TB >4 mg/dL and dilated CBD	18 (7.5)
▪ CBD stone on ultrasound/cross‐sectional imaging	120 (50.0)
▪ Ultrasound, *n* (%)	33 (27.5)
▪ CT, *n* (%)	80 (66.7)
▪ MRCP, *n* (%)	7 (5.8)
Clinical presentations to procedure time, days, median (IQR)	6 (2−15)
Choledocholithiasis on definitive Investigations, *n*/*N* (%)	
▪ EUS	117/138 (84.8)
▪ ERCP	209/219 (95.4)

ALT, alanine transaminase; AST, aspartate transaminase; CBD, common bile duct; CT, computed tomography; CVD, cardiovascular diseases; DLP, dyslipidemia; DM, diabetes mellitus; ERCP, endoscopic retrograde cholangiopancreatography; EUS, endoscopic ultrasound; HT, hypertension; MRCP, magnetic resonance cholangiopancreatography; TB, total bilirubin.

### Diagnostic performance of the ASGE 2019 high‐risk criteria

Table 2 summarizes CBD stone status based on the ASGE 2019 high‐risk criteria. Overall, 87.1% of patients had positive findings. Subgroup analysis showed CBD stone positivity rates of 90.2% for ascending cholangitis, 55.6% for TB >4 mg/dL with a dilated CBD, and 89.2% for CBD stones on imaging.

**TABLE 2:** Common bile duct stone status according to the American Society for Gastrointestinal Endoscopy 2019 high‐risk criteria.

	Positive CBD stone, *n* (%)	Negative CBD stone, *n* (%)
Overall criteria	209 (87.1)	31 (12.9)
Ascending cholangitis	92 (90.2)	10 (9.8)
TB >4 mg/dL and dilated CBD	10 (55.6)	8 (44.4)
CBD stone on US/cross‐sectional imaging	107 (89.2)	13 (10.8)

Abbreviations: CBD, common bile duct; TB, total bilirubin; US, ultrasound.

Table 3 presents the diagnostic performance of the ASGE 2019 high‐risk criteria for choledocholithiasis, including sensitivity, specificity, PPV, NPV, and accuracy with 95% CI. For each criterion, ascending cholangitis had a specificity of 67.7% and a PPV of 90.2%; TB >4 mg/dL with dilated CBD had a specificity of 74.2% and a PPV of 55.6%; and CBD stones on imaging had a specificity of 58.1% and a PPV of 89.2%.

**TABLE 3:** The diagnostic performance of individual American Society for Gastrointestinal Endoscopy 2019 high‐risk criteria.

ASGE 2019 high‐risk criteria	Sensitivity (%) (95% CI)	Specificity (%) (95% CI)	PPV (%) (95% CI)	NPV (%) (95% CI)	Accuracy (%) (95% CI)
Ascending cholangitis	44.02 (37.18–51.03)	67.74 (48.63–83.32)	90.20 (84.38–94.00)	15.22 (12.04–19.05)	47.08 (40.63–53.61)
TB >4 mg/dL and dilated CBD	4.78 (2.32–8.62)	74.19 (55.39–88.14)	55.56 (34.83–74.51)	10.36 (8.57–12.48)	13.75 (9.66–18.76)
CBD stone on US/cross‐sectional imaging	51.20 (44.21–58.15)	58.06 (39.08–75.45)	89.17 (84.20–92.71)	15.00 (11.26–19.71)	52.08 (45.56–58.55)

Abbreviations: ASGE, American Society for Gastrointestinal Endoscopy; CBD, common bile duct; CI, confidence interval; NPV, negative predictive value; PPV, positive predictive value; TB, total bilirubin; US, ultrasound.

The overall accuracy of each ASGE high‐risk criteria varied, with ascending cholangitis and CBD stone on US/cross‐sectional imaging showing intermediate accuracy at 47.08% (95% CI 40.63%–53.61%) and 52.08% (95% CI 45.56%–58.55%), respectively. On the other hand, TB >4 mg/dL with dilated CBD had a much lower accuracy of only 13.75% (95% CI 9.66%–18.76%).

## DISCUSSION

There is uncertainty regarding the optimal CBD stone threshold for treatment decisions. The ASGE 2010 guidelines recommend ERCP when the likelihood exceeds 50%,^7^ while the ASGE 2019 guidelines aim to raise this threshold and reduce diagnostic ERCP.^8^ This study found that the overall high‐risk ASGE 2019 criteria have a PPV of 87.1% for choledocholithiasis, based on the 33% disease prevalence. If the updated ASGE 2019 criteria were followed, clinical outcomes could significantly improve when selecting high‐risk patients for therapeutic ERCP, with only 13% requiring diagnostic ERCP.

Similar results were found in earlier studies evaluating the ASGE 2019 criteria's performance. Chandran et al. performed a retrospective cohort study of 744 patients suspected of choledocholithiasis who underwent ERCP. Individuals with a history of sphincterotomy or cholecystectomy were not recruited. Comparing the ASGE 2010 and 2019 criteria, they revealed an improvement in a PPV for CBD stone from 76.2% to 82.5% within the high‐risk group.^11^ Hasak et al. retrospectively identified 1098 patients with suspected choledocholithiasis who underwent ERCP and/or cholecystectomy with IOC.^12^ They found that the PPV of the ASGE 2019 high‐risk criterion was 86.3% (accuracy 70.4%), which was higher than the 2010 value of 82.5% (accuracy 60.1%). In the recently validated study by Silva‐Santisteban et al.,^13^ in which 359 patients suspected of having choledocholithiasis were prospectively enrolled based on ESGE and ASGE 2010 and 2019 criteria, choledocholithiasis detected by ERCP, EUS, or MRCP was used as the gold standard. They found that, in each high‐risk patient, choledocholithiasis was noted in 83.1% for the ESGE criteria and at a similar percentage for the ASGE 2019 (81.6%, P = 0.7) and 2010 criteria (79.1%, *p* = 0.3). The above data showed that the proportion of diagnostic ERCP was between 14 and 18%, consistent with the findings of our study.

When considering ERCP as a minimally invasive treatment for patients with ascending cholangitis related to a CBD stone or biliary tract obstruction, these patients should undergo ERCP with either CBD stone removal or internal stenting for urgent biliary drainage, coupled with antibiotic treatment.^14^ In most guidelines,^7^, ^8^, ^15^ including ASGE 2019, ascending cholangitis is the criterion indicating direct ERCP. The present study showed that of the 102 patients with ascending cholangitis who were sent for ERCP, 92 (90.2%) had a CBD stone, demonstrating that choledocholithiasis is still the major cause of biliary sepsis in our population. This is higher than previously reported in Western countries (28%–70%),^16^ and this may be because, in addition to cholesterol stones, many of our patients also had pigmented stones from underlying conditions such as biliary tract parasites or thalassemia. Thus, with a specificity of 68% (> 50% probability of choledocholithiasis), the current investigation demonstrated that ascending cholangitis is a strong predictor of choledocholithiasis and ERCP should be performed on patients with this condition. Data from an additional Asian study have supported this conclusion. A retrospective cohort study was conducted by Wong et al. of 142 patients who presented at the emergency room with the high‐risk ASGE 2019 criteria of choledocholithiasis. These patients underwent an EUS to confirm the presence of a CBD stone before receiving ERCP in the same session. The authors discovered that, with an adjusted OR of 2.28 (95% CI 1.01–5.15, *p* = 0.047), ascending cholangitis is a significant predictor for CBD stone.^17^ Although patients with ascending cholangitis due to underlying choledocholithiasis typically benefit from therapeutic ERCP with stone extraction, a small proportion may pass CBD stones spontaneously. Therefore, an EUS‐first approach before elective ERCP may be appropriate for patients with mild severity who respond well to initial medical management. This strategy allows time for stabilization, confirms CBD stones via EUS, and may reduce unnecessary diagnostic ERCP.

The other two high‐risk AGSE 2019 criteria, TB >4 mg/dL with dilated CBD and CBD stone on US/cross‐sectional imaging, raise concerns about the use of direct ERCP. Because spontaneous stone migration out of the CBD may occur in up to 20% of cases of choledocholithiasis,^18^ this obviates the need for ERCP in such patients. According to our findings, of the 138 patients who met these two high‐risk criteria and were sent for EUS first, 21 cases (15.2%) were able to avoid a diagnostic ERCP, since EUS did not detect a CBD stone or other biliary tract diseases, and it was assumed that they had passed a CBD stone. In terms of the diagnostic performance of each criterion by our analysis, TB >4 mg/dL with dilated CBD yields a specificity of 74.2% and PPV of 55.6%, whereas CBD stone on US/cross‐sectional imaging gives a specificity of 58.1% and PPV of 89.2%. The former criterion has a much lower likelihood of encountering a CBD stone (PPV of 55.6% vs. 89.2%) than the latter. Therefore, while a positive finding of a CBD stone on preprocedural imaging might justify proceeding directly to ERCP, in cases with the other criterion (TB >4 mg/dL with dilated CBD), in centers where an expert endoscopist in EUS is available, the approach whereby EUS confirms the existence of CBD stone before undergoing ERCP in the same endoscopic session is useful in avoiding diagnostic ERCP.

Previous studies that examined the performance characteristics of these individual high‐risk predictors revealed that TB >4 mg/dL with dilated CBD gives a specificity of 80.9%–96.8% and a PPV of 71.9%–95.4%, while a CBD stone on US/cross‐sectional imaging yields a specificity of 85.6%–99.6% and a PPV of 88.2%–99.2%.^11^, ^12^, ^19^, ^20^ This is higher than found in our current study. We postulated that the variations might be attributable to the prevalence of the disease, the type of research (prospective vs. retrospective), and the different confirmatory tests (EUS, MRCP, ERCP, or IOC) used in each study for CBD stones. Therefore, when relying on these two predictors, there is still room for debate on the ideal threshold for sending the patients directly for ERCP. As an example, the ESGE guideline only accepted ascending cholangitis and CBD stones identified on the initial US as indications for ERCP.^15^ Three studies have compared the high‐risk ASGE 2019 criteria with the high‐risk ESGE criteria for choledocholithiasis. The results are inconsistent; the studies by Wangchuk et al. and Silva‐Santisteban et al. found similar accuracy between the two guidelines, while the Jagtap et al. study found that the ESGE guideline better identified patients with choledocholithiasis.

Our study, along with previous research, shows that the ASGE 2019 guidelines offer acceptable diagnostic accuracy for choledocholithiasis. However, further data are needed to determine the optimal ERCP indications. Increasing the threshold for high‐risk categorization must balance the cost of reduced sensitivity and the need for EUS, MRCP, or IOC for intermediate‐risk patients. Strengths of our study include its prospective design, adherence to the protocol, and follow‐up for true negative results. To the best of our knowledge, this is the first study to examine EUS in tandem with ERCP for high‐risk patients without ascending cholangitis. Limitations include the potential difficulty of implementing this strategy in centers without EUS expertise and the small sample size of the TB >4 mg/dL with dilated CBD subgroup, which may have introduced confounding factors.

In conclusion, our study confirms that the ASGE 2019 criteria are effective for diagnosing choledocholithiasis. Early ERCP is recommended for patients with visible CBD stones or ascending cholangitis, while EUS can reduce unnecessary diagnostic ERCP for patients with TB >4 mg/dL and dilated CBD in centers with EUS expertise.

## CONFLICT OF INTEREST STATEMENT

None.

## ETHICS STATEMENT

Approval of the research protocol by an institutional review board: The study followed the Declaration of Helsinki's ethical guidelines, and the protocol was reviewed and approved by the ethics committee of Rajavithi Hospital (approval no. 026/2564).

## PATIENT CONSENT STATEMENT

Written informed consent was obtained from the patients prior to study enrollment.

## CLINICAL TRIAL REGISTRATION

The study was registered with the Thai Clinical Trials Registry (registration no. TCTR20210812001).
